# Partial molar pregnancy with hydrops fetalis causing intrauterine fetal demise: A case report

**DOI:** 10.1002/ccr3.8006

**Published:** 2023-09-30

**Authors:** Anup Panthi, Madhur Bhattarai, Shailendra Katwal, Sushmita Bhandari, Rituraj Baral, Madhav Bhusal, Bishal Khaniya

**Affiliations:** ^1^ Tribhuvan University Institute of Medicine Maharajgunj Nepal; ^2^ Dadeldhura Subregional Hospital Dadeldhura Nepal; ^3^ Fatima Jinnah Medical University Lahore Pakistan; ^4^ Devdaha Medical College Rupandehi Nepal; ^5^ Department of Obstetrics and Gynaecology Tribhuvan University, Institute of Medicine Maharajgunj Nepal

**Keywords:** gestational trophoblastic diseases, hydropic degeneration, hydrops fetalis, IUFD

## Abstract

**Key Clinical Message:**

Timely prenatal diagnosis, regular checkups, and comprehensive counseling are vital in preventing and managing complications in high‐risk pregnancies like partial molar pregnancy with hydrops fetalis.

**Abstract:**

A live singleton fetus with partial molar pregnancy is a rare condition. We report a case of partial mole with hydrops fetalis causing intrauterine fetal demise (IUFD) in the third trimester. Our case involves a 20‐year primigravid without prior antenatal checkups who presented to outpatient department at 31 weeks and 5 days of gestation with lower abdominal pain, backache, vaginal spotting, and decreased fetal movement. Ultrasound revealed partial mole, hydrops fetalis, and IUFD. The patient underwent induced delivery expelling a 1900 gm female fetus with no viability and a placenta containing 650 gm of molar tissue. Placental tissue with cystic component was confirmed as molar tissue by histopathological examination. She was discharged a few days afterward and had undetectable beta‐human chorionic gonadotropin levels after a month. Prenatal diagnosis, counseling, rigorous antepartum surveillance, and appropriate postpartum follow‐up are essential for the best possible mother and fetal outcomes.

## Graphical abstract

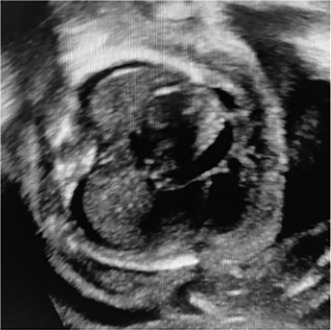

## 1 INTRODUCTION

Gestational trophoblastic diseases (GTD), refers to a collection of tumors with aberrant trophoblastic growth that is categorized as either hydatidiform moles, which have villi, or nonpolar trophoblastic malignant neoplasms, which do not have villi.^1^ Hydatidiform moles are excessively edematous immature placentas including the benign complete hydatidiform mole and partial hydatidiform mole.

A complete mole develops when a sperm fertilizes an empty ovum instead of a partial mole, which typically arises from dispermous fertilization of a normal haploid ovum that produces a triploid zygote or monospermic fertilization that duplicates the paternal haploid chromosome.^2^ Except in instances of multiple conceptions, partial molar pregnancy with a coexisting viable fetus (a condition sometimes called sad fetus syndrome) is extremely uncommon. About 0.005%–0.01% of all pregnancies result in a normal fetus with a partial molar pregnancy.^3^ Several factors may affect the outcome of the fetus in cases of partial molar pregnancy, such as the karyotype of the fetus, the size of the abnormal molar placenta, the speed of molar degeneration, the occurrence of fetal anemia, and other obstetric complications.^4^ We present a case diagnosed with partial molar pregnancy with hydrops fetalis causing intrauterine fetal demise (IUFD) at 31 weeks and 5 days of gestation with no previous antenatal care (ANC) visit. To the best of our knowledge, hydrops fetalis is not reported with partial molar pregnancy. This is probably the first association between partial mole, fetal death and hydrops to be reported.

## 2 CASE PRESENTATION

A 20‐year‐old female from a rural village in Far‐Western, Nepal reported to the outpatient department of a tertiary care hospital with amenorrhea for 8 months, lower abdominal pain, backache, vaginal spotting, and decreased fetal movement for 8 days. It was a planned and spontaneous pregnancy confirmed by a urine pregnancy test at home with no previous ANC visit at any health institution. This was a non‐consanguineous marriage and the husband's age was 28 years. At the time of presentation, she was prim‐gravida at 31 weeks and 5 days of gestation according to her last menstrual period. There was no history of iron and folic acid supplementation during her pregnancy. No family history of a genetic abnormality or difficult pregnancy was identified. She was a nonsmoker, nonalcoholic, and nonvegetarian by diet. There was no history of diabetes mellitus, thyroid disorders, or hypertension in the past.

**FIGURE 1:**
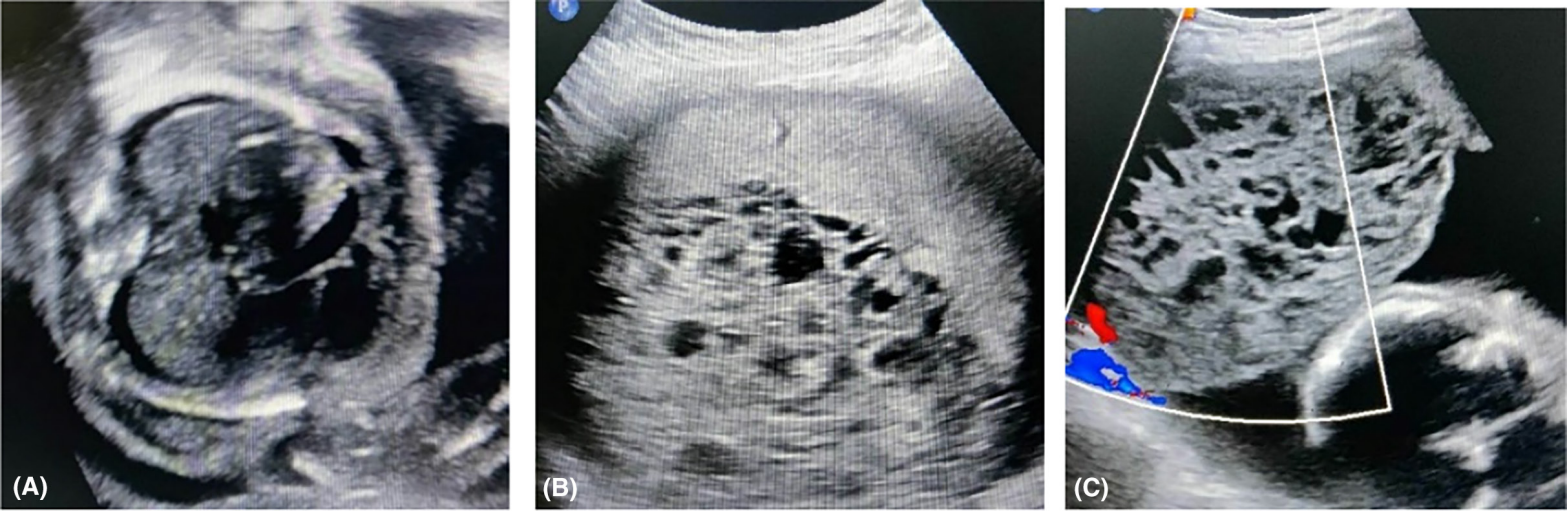
Ultrasound scans indicative of partial mole with hydrops fetalis in the dead fetus. Hypoechoic fluid (A) is seen in the dependent aspect of the bilateral pleural cavity and pericardial cavity. B and C show the part of the placental tissue with multiple cystic anechoic areas.

On physical examination, she was fair looking, alert, and well‐oriented to time, place, and person. Her blood pressure was 100/60 mmHg and a pulse of 70 beats/minute. Her abdomen was tender, and fundal height corresponded to 30 weeks with the cephalic presentation, and moderate contraction but fetal heart sound was absent. During her per vaginal examination, the external os was 2 cm dilated, the show was present and the membrane was intact. An ultrasound examination revealed an enlarged placenta with multiple anechoic areas in the anterior part of the placenta. There was minimal hypoechoic fluid noted in the pericardial and pleural cavities. Minimal ascites were also noted. No fetal cardiac activity was seen. No flow was seen in color and spectral Doppler study of the middle cerebral artery suggestive of fetal death (Figure 1A). We also noted evidence of a partial mole indicated by a mass resembling the bunch of grapes (hydropic degeneration) on the anterior fundus with a measurement of 11 cm by 8 cm bulging into the amniotic cavity and compressing the fetus. (Figure 1B,C).

**FIGURE 2:**
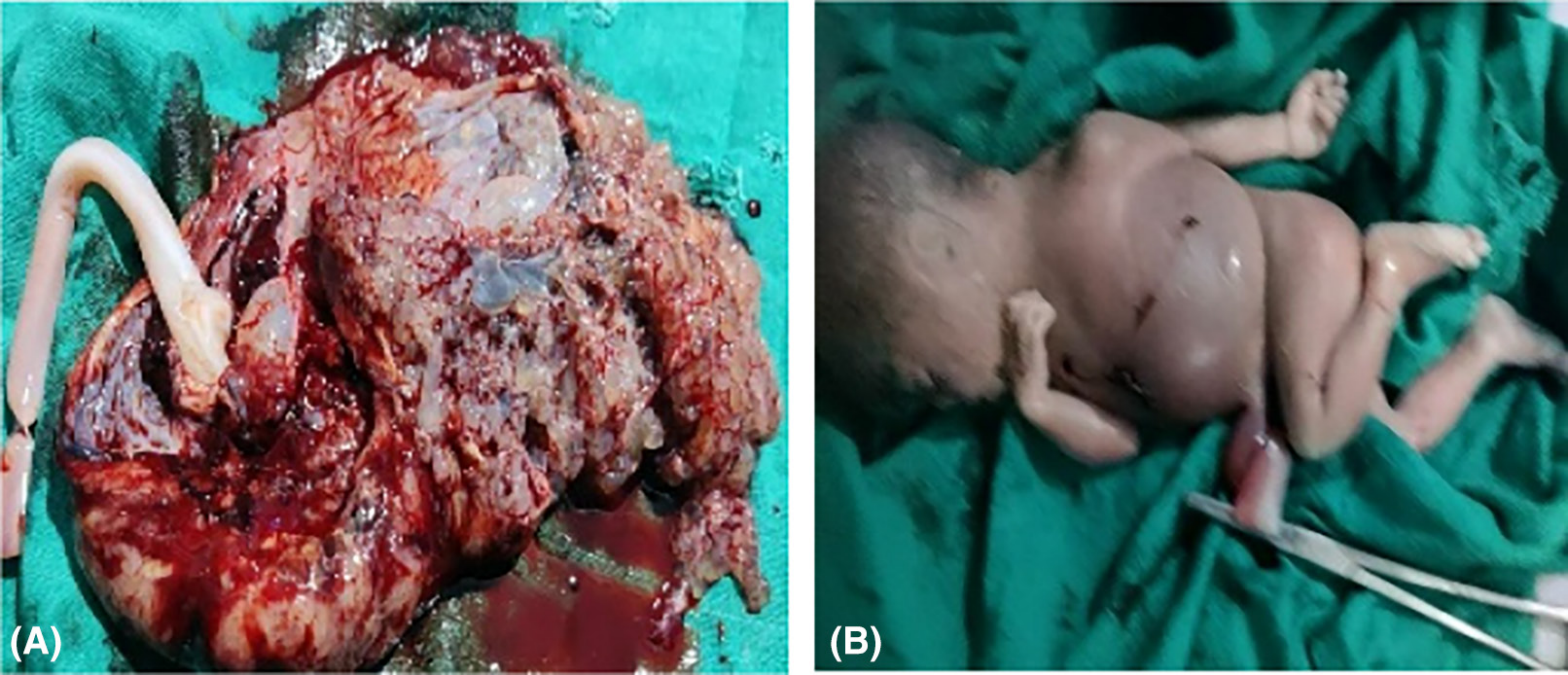
Photograph of the expelled placental tissue with multiple grape‐like vesicles (A) and dead fetus with a distended abdomen (B).

Lab investigations at admission were normal except for elevated beta human chorionic gonadotropin (β‐hCG) level (582,764 mIU/mL). After this, an abortion was induced with misoprostol given vaginally. After 4 hours a dead female fetus was delivered vaginally weighing 1900 gm with an APGAR score of 0 at 1 and 5 minutes, respectively. After this, she expelled a molar tissue weighing 650 gm along with the placenta (Figure 2A,B).

**FIGURE 3:**
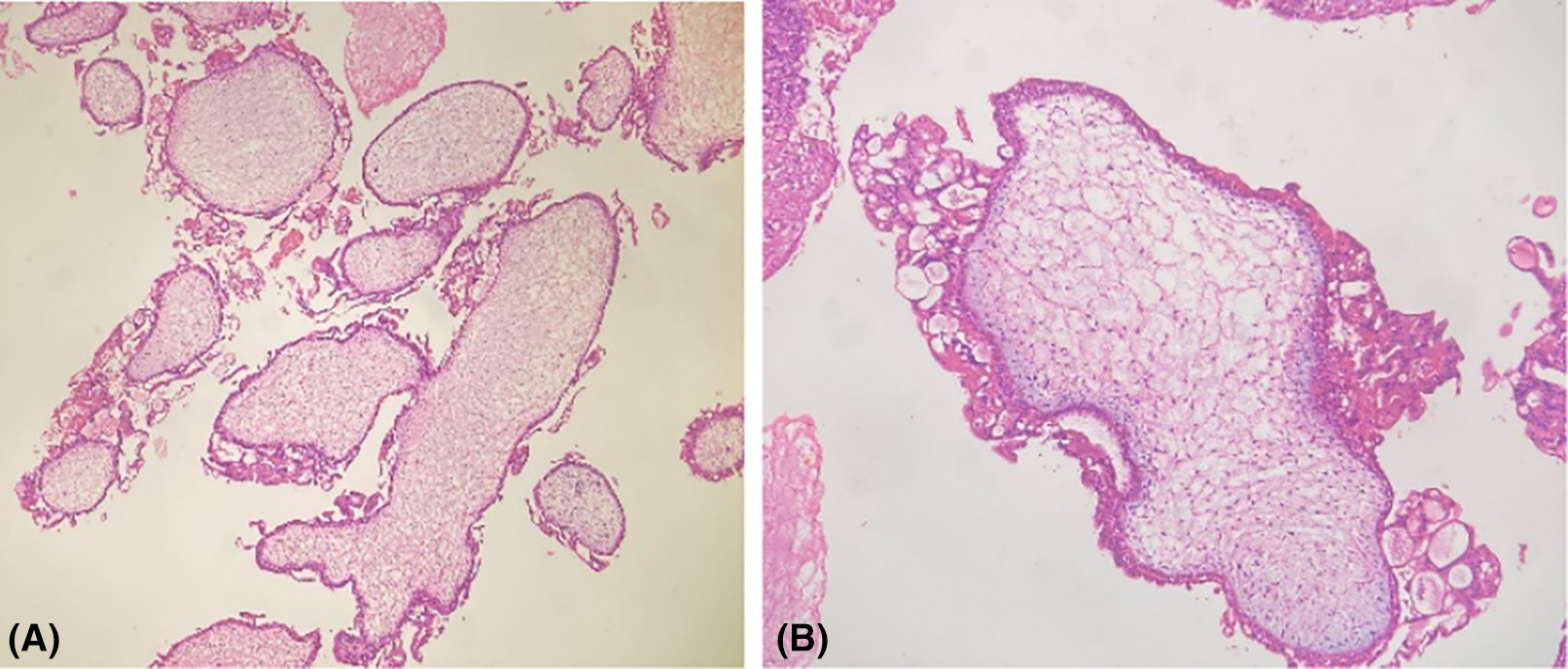
Low power view shows heterogeneous population of chorionic villi (A) and chorionic villi showing polar proliferation of trophoblastic cells (B).

The molar tissue was sent for histopathological examination which shows heterogeneous population of chorionic villi (Figure 3A) with polar proliferation of trophoblastic cells (Figure 3B).

After delivery intramuscular syntocin 10 units were given, and Foley's catheterization was done. Injection tramacet 1 gm, injection ceftriaxone 1 gm, and injection of metronidazole 500 gm were given intravenously immediately. Vaginal packing was done with three delivery pads and bleeding was stopped. Approximately 1 day after the patient was discharged home, we noted a β‐hCG level of 200,000mIU/mL. Upon discharge, we instructed the patient to follow‐up with serial measurements of her β‐hCG levels. After a month, her β‐hCG level was undetectable. β‐hCG level was undetectable for three consecutive visits in our center.

## 3 DISCUSSION

The incidence of molar pregnancy is in increasing trend with decreasing maternal age below 20 years as well as are with increasing maternal age of patients over 35 years.^5^ Molar pregnancies with a coexistent live fetus occur in three variations. The most common variant includes a twin pregnancy with one fetus being healthy and the other being a complete mole followed by a twin pregnancy harboring a healthy fetus/placenta with a partial mole, and the rarest variant is a healthy single fetus with a partial molar placenta,^6^ similar to how it was with our patient. Ultrasound is the primary diagnostic modality of partial molar pregnancy. It reveals placental changes, placenta insufficiency, fetal malformation, abnormal growth, and oligohydramnios. After ultrasound, single fetus with a partial molar placenta, dichorionic twins of normal fetus and complete mole, placental mesenchymal dysplasia (PMD), hydropic abortion were made as differentials. Increased trophoblastic proliferation on histopathology, increased β‐hCG, and presence of normal fetus ruled out the differentials with final diagnosis of single fetus with partial mole.

Definitive diagnosis is done by cytogenetic analysis via amniocentesis or chorionic villus sampling.^7^ The majority of partial hydatidiform moles that coexist with fetal tissue or an abnormal fetus are caused due to dispermy and have a triploid karyotype. However karyotyping was not done in our case. In one of the systemic reviews by Mangla et al.^3^ partial molar pregnancy associated with the live birth rate was 56.8%, whereas intrauterine death was reported in 20% of cases. The cause of intrauterine death associated with partial molar pregnancy is fetal distress with severe anemia, congenital anomaly, and maternal complications such as preeclampsia, thyrotoxicosis, and vaginal bleeding. Bleeding in the first or second trimester of pregnancy is one of the most common complications in such cases. Bleeding at any time may prove life‐threatening for the mother, thereby necessitating urgent delivery.^8^ In molar pregnancy, trophoblastic tissue results in excessive production of circulating antiangiogenic factors like fms‐like tyrosine kinase receptor 1, which plays a pathogenic role in GHTN.^9^ Though mothers are expected to develop GHTN/PE due to hydatidiform mole, vaginal bleeding is still the most prevalent presenting symptoms. Anemia, preeclampsia, hyperemesis, and clinical hyperthyroidism are substantially less common as presenting symptoms among patients.^10^

**FIGURE 4:**
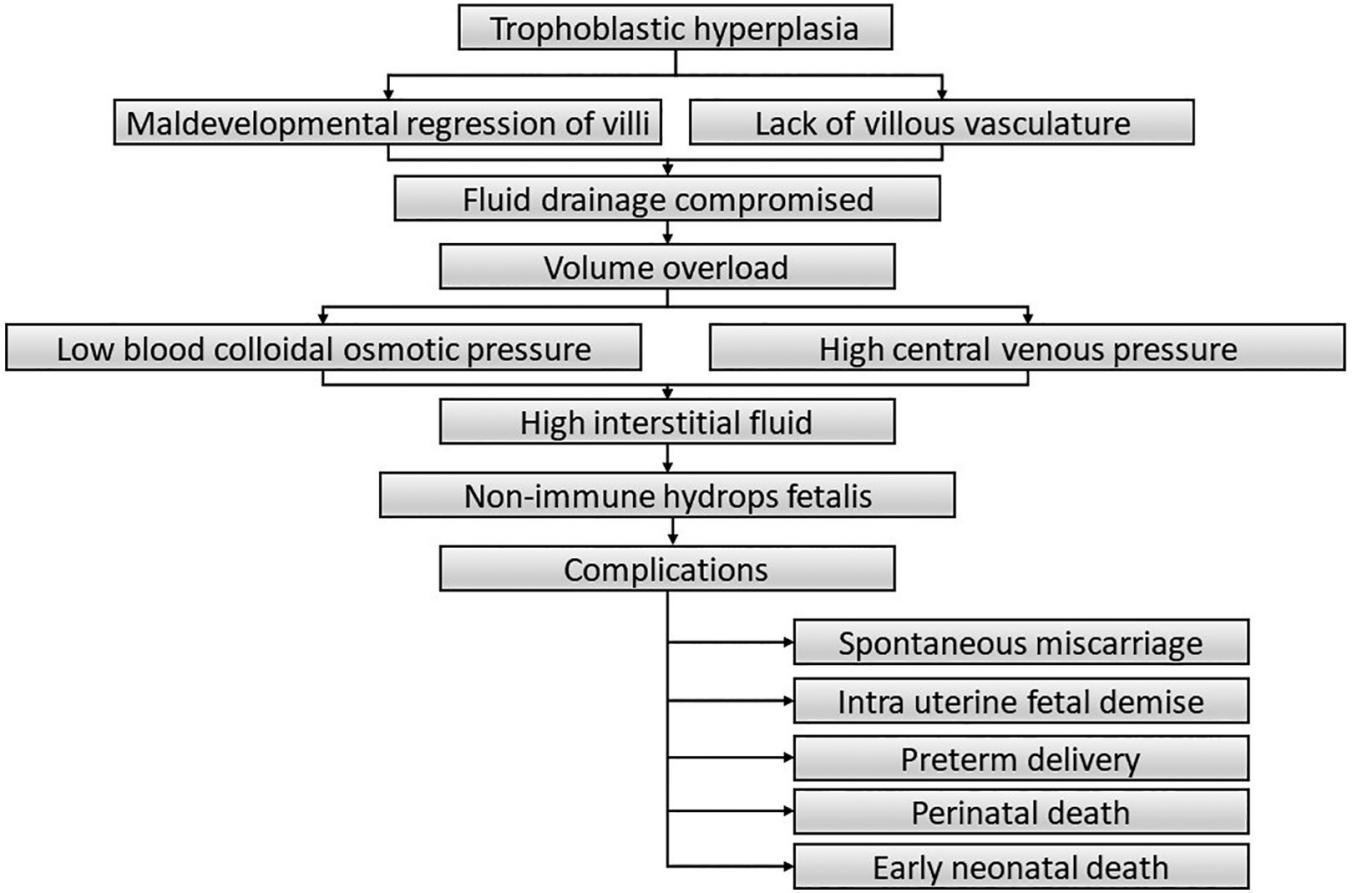
Flowchart showing possible pathogenesis of IUFD in partial mole with hydrops.

One in every 1700 babies is estimated to have hydrops fetalis, which is defined by an abnormal buildup of fluid in two or more fetal serous cavities, including ascites, pleural effusion, pericardial effusion, and skin edema. Rhesus isoimmunization is the most common cause of hydrops fetalis but the etiology has switched to nonimmune causes after the introduction of Rhesus (D) immune globulin. Nonimmune fetal hydrops (NIHF) is frequently associated with an increased risk of preterm labor, intrauterine fetal demise (IUFD), and newborn morbidity and mortality.^11^ It occurs because of various causes that interferes with fetal body's ability to manage fluid. Maternal IgM and IgG serology are obtained for TORCH and parvovorus B19 which were nonreactive and major cause of infection were ruled out. Hematological investigations of cord blood were also normal. The possible explanation for partial mole, hydrops, and intrauterine fetal demise is shown in Figure 4.

In this unbooked case, the patient presented to our institute as a primigravida at 31 weeks and 5 days of gestation, based on her last menstrual period. Due to a lack of awareness, difficult topography, poverty, and limited access to healthcare facilities, she had not received any antenatal checkups. As a result, prenatal diagnosis, appropriate counseling, and rigorous antepartum surveillance could not be provided to the patient. However, after her presentation, we provided postnatal counseling to prevent a similar episode in her next pregnancy. It is important to note that if these interventions had been implemented at the appropriate time, she could have been managed earlier and received necessary counseling, thereby preventing complications.

Partial molar pregnancy with a viable fetus is a rare disease with a difficult diagnosis. The optimum technique to address a hydatidiform mole with a coexisting live fetus is uncertain. Clinicians are asked to share their unique experiences so that recommendations for the care and prenatal counseling of pregnancies with partial mole and coexistent fetuses can be generated.^7^

## 4 CONCLUSION

This case report highlights the rare condition of partial molar pregnancy with hydrops fetalis causing IUFD. Prenatal diagnosis, counseling, thorough antepartum surveillance, and proper postpartum follow‐up are critical for optimal mother and fetal outcomes. In such a pregnancy, an obstetrician, gynecologic oncologist, and neonatologist should be involved in the care.

## AUTHOR CONTRIBUTIONS

**Anup Panthi:** Conceptualization; writing – original draft; writing – review and editing. **Madhur Bhattarai:** Writing – original draft; writing – review and editing. **Shailendra Katwal:** Writing – original draft; writing – review and editing. **Sushmita Bhandari:** Writing – review and editing. **Rituraj Baral:** Writing – review and editing. **Madhav Bhusal:** Writing – review and editing. **Bishal Khaniya:** Writing – review and editing.

## ACKNOWLEDGMENTS

None.

## CONFLICT OF INTEREST STATEMENT

Authors have no conflict of interest to declare.

## CONSENT

Written informed consent was obtained from the patient to publish this report in accordance with the journal's patient consent policy.

## Data Availability

All the required information is available in the manuscript itself.
